# Expression of c-Fos in the rat retrosplenial cortex during instrumental re-learning of appetitive bar-pressing depends on the number of stages of previous training

**DOI:** 10.3389/fnbeh.2013.00078

**Published:** 2013-07-04

**Authors:** Olga E. Svarnik, Alexandra I. Bulava, Yuri I. Alexandrov

**Affiliations:** ^1^V.B. Shvyrkov Laboratory of Neural Bases of Mind, Institute of Psychology of Russian Academy of SciencesMoscow, Russia; ^2^Laboratory of Systems Neurophysiology and Neuronal Interfaces, Neuroscience Department, Kurchatov NBICS-CenterMoscow, Russia

**Keywords:** learning, c-Fos, behavior, history of training, bar-press

## Abstract

Learning is known to be accompanied by induction of c-Fos expression in cortical neurons. However, not all neurons are involved in this process. What the c-Fos expression pattern depends on is still unknown. In the present work we studied whether and to what degree previous animal experience about Task 1 (the first phase of an instrumental learning) influenced neuronal c-Fos expression in the retrosplenial cortex during acquisition of Task 2 (the second phase of an instrumental learning). Animals were progressively shaped across days to bar-press for food at the left side of the experimental chamber (Task 1). This appetitive bar-pressing behavior was shaped by nine stages (“9 stages” group), five stages (“5 stages” group) or one intermediate stage (“1 stage” group). After all animals acquired the first skill and practiced it for five days, the bar and feeder on the left, familiar side of the chamber were inactivated, and the animals were allowed to learn a similar instrumental task at the opposite side of the chamber using another pair of a bar and a feeder (Task 2). The highest number of c-Fos positive neurons was found in the retrosplenial cortex of “1 stage” animals as compared to the other groups. The number of c-Fos positive neurons in “5 stages” group animals was significantly lower than in “1 stage” animals and significantly higher than in “9 stages” animals. The number of c-Fos positive neurons in the cortex of “9 stages” animals was significantly higher than in home caged control animals. At the same time, there were no significant differences between groups in such behavioral variables as the number of entrees into the feeder or bar zones during Task 2 learning. Our results suggest that c-Fos expression in the retrosplenial cortex during Task 2 acquisition was influenced by the previous learning history.

## Introduction

It has been widely shown that learning situations induce immediate early gene (IEG) expression in brain neurons of various species (Kaczmarek and Chaudhuri, 1997; Herdegen and Leah, 1998; Tischmeyer and Grimm, 1999; Clayton, 2000; Miyashita et al., 2008). One IEG, *c- fos* gene, might be used as a cellular marker of learning-related neuronal plasticity (Anokhin and Rose, 1991; Rylski and Kaczmarek, 2004; Lyons and West, 2011). Animals' brains in the absence of learning situations (in home caged controls or in animals executing an over-trained skill) are characterized by a low number of c-Fos positive neurons (Anokhin et al., 2001; Svarnik et al., 2005). Only some structures show an elevated number of c-Fos positive neurons after learning. Distribution of c-Fos positive neurons among brain structures seems to depend on the learning paradigm. Fear learning in rodents induced c-Fos in the auditory, medial prefrontal, orbitofrontal, intralimbic neurons, amygdala, anterior hypothalamus, brainstem monoaminergic nuclei and periaqueductal gray neurons (Trogrlic et al., 2011; Peter et al., 2012; Tulogdi et al., 2012). A spatial memory task elicits *c-fos* expression in the hippocampus and the medial prefrontal cortex (Feldman et al., 2010; Lopez et al., 2012). A novelty recognition task in mice activated c-Fos expression in the basolateral amygdala, the paraventricular hypothalamic nucleus, the suprapyramidal blade of the dentate gyrus and the medial prefrontal cortex (Castilla-Ortega et al., 2012).

Learning induces IEG expression not in the whole structures but only in subsets of neurons. About 70% of pallial HVC nucleus and robust nucleus of arcopallium neurons in zebra finches were c-Fos positive after food aversion learning (Tokarev et al., 2011). New environment exploration induced *arc* (another activity-related IEG) in 18% of CA3 hippocampal neurons, in 35% of CA1 hippocampal neurons and in 2% of granule cells in the dorsal blade of the dentate gyrus of the hippocampus (Kubik et al., 2007). About 20% of neurons were c-Fos positive in the rat retrosplenial cortex after instrumental task acquisition (Svarnik et al., 2005). It has been shown previously that neuronal firing during food-acquisition behavior depended on training strategy (Gorkin and Shevchenko, 1996; Alexandrov, 2008). Then it might be suggested that such behavior-related neurons retained previous training history by means of c-Fos induction at every re-learning that occurred previously. In the present work we studied whether and to what degree previous animals' experience about appetitive bar-pressing Task 1 influence neuronal expression of c-Fos protein in the retrosplenial cortex during acquisition of appetitive bar-pressing Task 2. The retrosplenial cortex was selected for this kind of analysis because previous studies using neuronal recording methods indicated that many retrosplenial neurons were specifically activated during bar-pressing appetitive task (Aleksandrov et al., 1997; Svarnik et al., 2005).

## Materials and methods

Female Long-Evans hooded rats (250–300 g) were housed individually in a colony room. Rats were maintained in a normal 12:12 LD cycle, allowed *ad libitum* water, but deprived of food and maintained throughout the experiment at a level so that their weight loss did not exceed 15% of the free-feeding body weight. All animal procedures in these studies were in accordance with the National Institutes of Health “Guidelines for the Care and Use of Animals for Experimental Procedures,” which were approved by the Russian Academy of Sciences. The number of animals used and their suffering were minimized.

All behavioral training took place in an instrumental chamber of 40 × 40 × 50 cm. The chamber was fitted with two automated plastic feeders in the corners and two bars located in the opposite corners. A button controlled by an experimenter was located outside of the cage and allowed filling a required feeder at any time. The rat's behavior throughout training was video recorded for off-line analysis.

Training was conducted daily in 30-min sessions. Animals were progressively shaped across days to bar-press for food on the left side of the experimental cage—Task 1 (Kelly and Deadwyler, 2003; for details see Svarnik et al., 2005). Bar-pressing behavior was shaped by nine stages (“9 stages” group, *n* = 4), five stages (“5 stages” group, *n* = 5) or one intermediate stage (“1 stage” group; *n* = 5). As intermediate stages we rewarded approaching a feeder (for “1 stage” group); approaching a feeder, turning away from a feeder, approaching the middle of cage side, approaching a bar (for “5 stages” group); approaching one or another feeder, turning away from one or another feeder, approaching the middles of cage sides, approaching one or another bar (for “9 stages” group). A new behavioral event was introduced at the beginning of the following session. Every day animals had to learn a newly rewarded behavioral event until they learned to bar-press on the left side of the experimental chamber—Task 1. The implemented difference between the groups was the number of re-learnings (depending on the number of intermediately rewarded stages) up to the acquisition of Task 1. After all animals acquired Task 1, they had to practice it for 5 days. Task 1 was considered to be acquired if an animal performed five presses in a row. If an animal did not reach this criterion, this pressing stage was repeated on the next day, and the length of shaping was prolonged for a whole series. The total number of days spent in the experimental cage was 10–14 and also depended on the animals' behavior during the first day of training. If an animal did not start eating from a feeder during the first day of training, this first stage was repeated on the next day, and the length of shaping was prolonged for a whole series. Each series consisted of six animals (six brain sections were fitted on one glass for immunohistochemical processing) from at least three different groups (including one control animal in each series). During the last experimental session the bar and feeder on the left, familiar side of the chamber were turned off, and the animals were allowed 30 min to learn a similar instrumental task at the opposite, rewarded side of the chamber using another pair of a bar and a feeder (Task 2). Animals of a control group (*n* = 3) were kept in their home cages and were sacrificed at the same time as trained animals.

Off-line analysis of behavior was performed by using a custom made EasyTrack software. Tracking was based on the “center of gravity” of animals' dark contour. We defined zones of interest around each feeder and lever in such a way that an animal taking food from a feeder or pressing a lever from any position around it would be considered to be located in these zones. Behavioral variables during the last experimental session included the number of entries into zones of interest (the unrewarded feeder zone, the unrewarded bar zone, the rewarded feeder zone, the rewarded bar zone), total distance traveled, mean speed and maximum speed. The Kruskal–Wallis test (χ^2^ median test) and Mann–Whitney rank sum test for pairwise comparisons were used for analysis of behavioral variables between the groups. All statistical tests were performed in Statistica 5.0.

Seventy five minutes after the last experimental session animals were exposed to diethyl ether for five minutes and decapitated. Their brains were removed and frozen for analysis. Coronal 20 μm cryostat brain sections were taken through a part of the retrosplenial agranular (RSA) cortex from 4.0 to 5.0 mm posterior to bregma (Paxinos and Watson, 1997). We collected one out of five brain sections through the selected length (10 sections per brain). We chose this part of the cortex because it was shown earlier that this area contained a high percentage of bar-pressing task related neurons (Aleksandrov et al., 1997; Svarnik et al., 2005). The sections prepared for immunohistochemistry were dried overnight and fixed in 4% paraformaldehyde in 0.1 M phosphate-buffered saline (PBS), pH7.4, for 15 min. Fixed sections were washed (3 × 5 min) in 0.1 M PBS and placed into a blocking solution (2.5% normal serum/0.1 M PBS) for 30 min. The sections were then incubated in c-Fos rabbit polyclonal antibody (Ab-5, “Calbiochem”, USA), diluted 1:2000 with 0.1 M PBS, for 18 h at room temperature. The sections were washed (6x5 min) with 0.3% Triton X-100 in 0.1 M PBS, and incubated with biotinylated goat anti-rabbit secondary antibody (“Vector Laboratories”, USA) diluted 1:400 in PBS for 2 hr. They were then washed (5 × 5 min) and processed with the 1% streptavidin-biotin complex (“Vector Laboratories”, USA) for 1 h. After 4 × 5 min washes the sections were placed in a solution of 0.06% diaminobenzidine (DAB, Sigma, USA) and 0.003% H_2_0_2_ for 6 min. The sections were then washed in tap water, dehydrated and coverslipped with the mounting medium.

**Figure 1:**
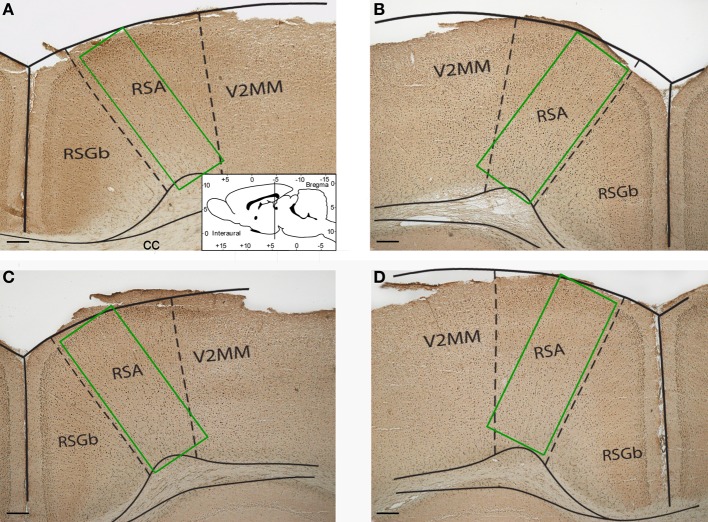
**Representative photomicrographs showing sample areas of the retrosplenial cortex of control (A), “1 stage” (B), “5 stages” (C), and “9 stages” (D) group animals**. Coronal sections of 20 μm thickness. Scale bar = 200 μm. RSA, retrosplenial agranular cortex; RSGb, retrosplenial granular b cortex; V2MM, secondary visual cortex, mediomedial area; cc, corpus callosum.

Images of the retrosplenial cortex were digitized at 4x magnification under an Olympus BX-50 microscope (Japan) by WV-CP230 camera (Panasonic, Japan) and analyzed using Image-Pro Plus (Media Cybernetics, USA). The number of Fos-positive cells was counted in sample areas of retrosplenial cortex (Figure 1). The counting was performed by an investigator blind to the experimental group assignment of the animals. The Kruskal–Wallis test (χ^2^ criterion) and Mann–Whitney rank sum test for pairwise comparisons were used to compare the number of Fos-positive neurons between the groups. All statistical tests were performed in Statistica 5.0.

A probability level of *p* = 0.05 was accepted as statistically significant.

## Results

**Figure 2:**
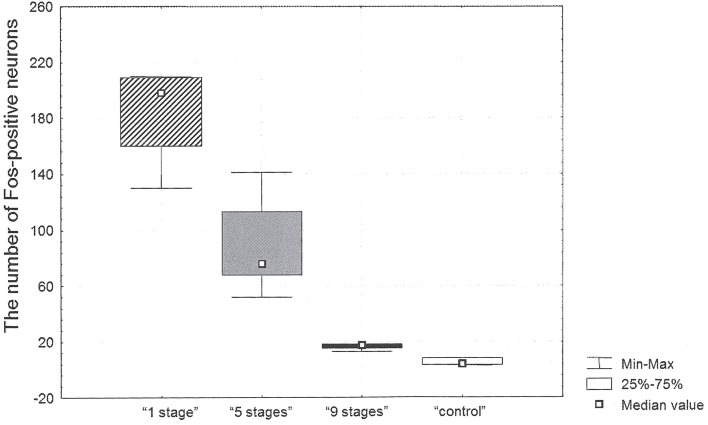
**The number of c-Fos positive neurons in the retrosplenial cortex after Task 2 acquisition in animals of “1 stage” group, “5 stages” group, “9 stages” group and “control” group**.

The highest number of c-Fos positive neurons was found in the retrosplenial cortex of “1 stage” animals (198 [160; 209] per mm^2^, all data presented as median [25th percentile; 75th percentile]) as compared to the other groups. The numbers of c-Fos positive neurons in the studied area equaled 76 [68; 113] mm^2^ in “5 stages” animals, 18 [15; 18] per mm^2^ in “9 stages” animals and 4 [3; 8] per mm^2^ in home cage control animals (see Figure 2).

There were significant differences in the number of c-Fos positive neurons between groups (Kruskal–Wallis χ^2^ = 9.2; *df* = 2; *p* = 0.01). The number of c-Fos positive neurons in the cortex of “9 stages” animals was significantly higher than the one in quiet home cage control animals (Mann–Whitney *z* = 2.12; *p* = 0.03). The number of c-Fos positive neurons in this cortex of “5 stages” animals was significantly higher than the one in “9 stages” group animals (Mann–Whitney *z* = −2.45; *p* = 0.01). The number of c-Fos positive neurons in the cortex of “1 stage” animals was significantly higher than in “5 stages” group animals (Mann–Whitney *z* = 2.40; *p* = 0.02).

We also assessed behavioral variables during the last 30-minute session when animals acquired Task 2 using the second pair of a bar and a feeder on the second side of the experimental cage (see Table 1). There were no significant differences between the groups in total distance traveled (Kruskal–Wallis χ^2^ = 3; *df* = 2; *p* = 0.22), mean speed (Kruskal–Wallis χ^2^ = 1.4; *df* = 2; *p* = 0.49), maximum speed (Kruskal–Wallis χ^2^ = 1.4; *df* = 2; *p* = 0.49), the number of entries into “ unrewarded feeder” zone (Kruskal–Wallis χ^2^ = 3; *df* = 2; *p* = 0.22), the number of entries into “ rewarded feeder” zone (Kruskal–Wallis χ^2^ = 0.4; *df* = 2; *p* = 0.81), the number of entries into “ unrewarded bar” zone (Kruskal–Wallis χ^2^ = 1.4; *df* = 2; *p* = 0.49), or the number of entries into “ rewarded bar” zone (Kruskal–Wallis χ^2^ = 3.6; *df* = 2; *p* = 0.17).

**Table 1:** **Behavioral variables during Task 2 acquisition in animals of “1 stage” group, “5 stages” group and “9 stages” group**.

**Behavioral variable**	**Groups**	**Median**	**25,000th percentl**	**75,000th percentl**
Total distance traveled (m)	“1 stage”	130.69	130.62	194.43
	“5 stages”	113.12	103.02	113.77
	“9 stages”	186.39	138.63	213.47
Mean speed (cm/s)	“1 stage”	7.28	7.26	10.82
	“5 stages”	6.30	5.69	7.36
	“9 stages”	10.37	7.68	11.87
Maximum speed (cm/s)	“1 stage”	161.61	148.52	191.32
	“5 stages”	176.36	149.29	492.77
	“9 stages”	212.52	183.51	219.58
Number of entries into “unrewarded feeder” zone	“1 stage”	63	46	92
	“5 stages”	71	65	88
	“9 stages”	41	32	72
Number of entries into “rewarded feeder” zone	“1 stage”	177	171	226
	“5 stages”	113	97	174
	“9 stages”	258	127	390
Number of entries into “unrewarded bar” zone	“1 stage”	107	79	129
	“5 stages”	87	59	90
	“9 stages”	77	56	94
Number of entries into “rewarded bar” zone	“1 stage”	172	166	192
	“5 stages”	104	63	148
	“9 stages”	228	105	352

**Figure 3:**
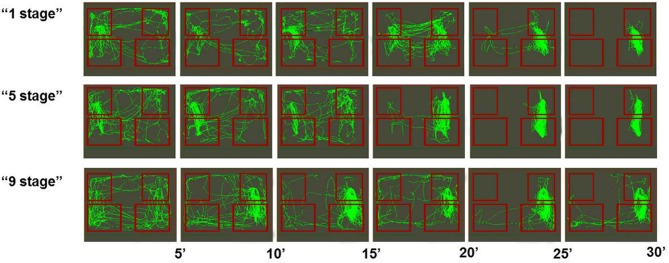
**Movement paths of representative rats (from all three groups) that successfully learned Task 2**. Rectangles represent feeder zones (at the top) and bar zones (at the bottom). Rewarded locations are on the right side of the experimental cage, and unrewarded locations are on the left side of the experimental cage. Movement paths are summarized for every 5-min period of the 30-min experimental session.

**Figure 4:**
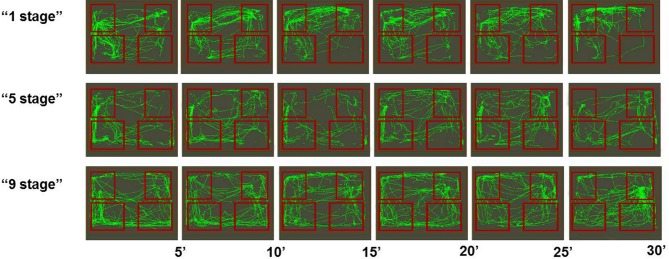
**Movement paths of representative rats (from all three groups) that did not learned Task 2 successfully**. Rectangles represent feeder zones (at the top) and bar zones (at the bottom). Rewarded locations are on the right side of the experimental cage, and unrewarded locations are on the left side of the experimental cage. Movement paths are summarized for every 5-min period of the 30-min experimental session.

Among all groups there were more successful (see Figure 3) and less successful learners of the second task (see Figure 4). Animals were considered to be unsuccessful in Task 2 if they did not perform at least five presses in a row during the last experimental session. One animal in each group was unsuccessful in Task 2. Successful learners performed Task 2 on the second, rewarded side of the chamber (right side on the picture) at least during the last half of the session. We used two criteria for measuring success. The first one was the percentage of rewarded bar zone entrances out of the total number of any zone entrances. The second one was the ratio between the number of rewarded bar zone entrances and unrewarded bar zone entrances. These two parameters were highly correlated (Spearman *Rs* = 0.98; *p* < 0.0001). The success in Task 2 was positively correlated with the number of c-Fos positive neurons in the retrosplenial cortex of “1 stage” group animals (Spearman *Rs* = 1; *p* < 0.0001), but not of “5 stages” or “9 stages” (*p* > 0.05).

## Discussion

Previous studies using neuronal recording methods indicated that many neurons of the retrosplenial cortex were specifically activated during bar-pressing appetitive task (Aleksandrov et al., 1997; Svarnik et al., 2005). Such specific activities are probably acquired during task learning through the mechanism of IEG activation in those neurons. The key finding of this study was that an increase in c-Fos expression in the rat retrosplenial cortex after Task 2 was related to the way the animals learned previous Task 1. Animals that acquired the first task in one stage had more c-Fos positive neurons than animals that acquired it in five stages, which in turn had more Fos-positive neurons than animals that acquired it in nine stages. In contrast, the behavior of animals of all groups during Task 2 learning did not differ significantly. During the first 10-min period of Task 2 acquisition animals of all groups performed Task 1 stereotyped behavioral sequences (bar-pressing on the left side of the experimental cage), which were not rewarded anymore. The middle period of this session was characterized by a large number of entrances into the rewarded feeder zone. During the last period of the session animals of all groups were mostly engaged in bar-pressing on the rewarded side of the experimental chamber, which was manifested by a large number of entrances into the rewarded feeder zone and the rewarded bar zone. Thus c-Fos expression was not directly associated with animals' ongoing activity. Moreover, not all animals in each group acquired Task 2, so their activity during the last session had different levels of success, but still the effect of previous training history on Fos expression seemed to be greater. Individual behavioral differences are of great interest and have been broadly discussed in animal learning studies (Sandi and Touyarot, 2006; Lehner et al., 2008, 2009; Schulz and Korz, 2010; Gökçek-Saraç et al., 2012), but their relations to neuronal functioning (including Fos expression) peculiarities are still to be determined in further experiments.

C-Fos is often considered to be an indicator of neuronal activity (Morgan and Curran, 1989; Hoffman et al., 1993; Coggeshall, 2005). More generally c-Fos is thought to map neuronal populations that respond to some kind of stimulation (Hunt et al., 1987; Barth et al., 2004). At the cellular level c-Fos expression was shown to require action potential firing, but not synaptic activity (Schoenenberger et al., 2009). However, other findings show that neuronal activity itself is not sufficient for c-Fos induction. For example, it has been shown that the number of Fos-positive neurons in animals performing overtrained behavior did not differ significantly from home caged control animals (Kleim et al., 1996; Anokhin et al., 2001). Additionally as we showed earlier for animals trained to bar-press, the percentage of c-Fos positive neurons was not directly related to the percentage of active neurons during task performance (Svarnik et al., 2005). It has been also shown that an increase in firing activity alone is not sufficient for c-Fos induction (Luckman et al., 1994). All these findings imply that c-Fos is rather “a cellular marker of neural activity and neuroplasticity” (VanElzakker et al., 2008) but not of firing action potentials alone. It has been recently noted that if depolarization *per se* induces c-Fos expression, c-Fos should have been detected “in millions of neurons throughout the brain under basal conditions”, which is not the case (Kovacs, 2008). All of these imply that there are complex relationships between synchronous firing of neurons and c-Fos induction (Labiner et al., 1993). C-Fos seems to be induced not by firing action potentials, but by changes in existing firing pattern or changes in specifically patterned activity of neurons. It goes along with the fact that c-Fos is inducted by seizures (Morgan et al., 1987), when neuronal firing happens in all possible, not regular combinations or patterns. It has been shown that c-Fos might be induced by novelty or mismatch between an expected and actual situation (Anokhin and Sudakov, 2003; VanElzakker et al., 2008) or between a need and possibility for its satisfaction (Aleksandrov, 2006). In the present study we showed that c-Fos expression was rather associated with changes in previously acquired neuronal groups. It seems that c-Fos might be induced by firing of neurons in new combinations, underlying new combinations of behavioral sequences. In the case of new learning or re-learning two things are happening to already existing neuronal groups at the same time: their activation and their reorganization due to a new pattern of neuronal activity. Or, in other words, every learning is reconsolidation of pre-existed memory (Tse et al., 2011; Dudai, 2012). In this sense, our case of c-Fos induction seems to be similar to the one found under reactivation of previous experience, for example, during sleep (Marrone et al., 2008).

C-Fos expression after Task 2 acquisition turned out to be higher in those groups of animals that experienced fewer intermediate stages of learning during Task 1 acquisition. Bar-press shaping through intermediate stages meant that animals went through several stages of learning followed by extinctions. It was shown that extinction did not erase the existing memory but formed a new memory circuit (Milad and Quirk, 2002). Animals that experienced more extinction stages probably formed more neuronal groups related to the studied behavior in the given experimental chamber. We showed earlier that animals which learned to pedal-press by a step-by-step shaping procedure had more task-related neurons than animals which learned the same task in one step (Alexandrov, 2008). Reactivating previously formed behavior over and over again these animals reorganized their previous experience to a higher degree. Such reorganization may underlie the ability which is called “learning to learn”. These animals also had more opportunities to perform orienting or trial behavior in the experimental chamber during previous learning. All these circumstances might cause c-Fos expression in a fewer percentage of neurons during subsequent re-learning. Having more differentiated experience might mean less possibility of reorganization of this experience during re-learning. At the same time the current learning situation can also influence c-Fos distribution. We showed that at least in “1 stage” animals success in the current task acquisition correlated with c-Fos expression. Both new experience formation and old experience reorganization possibly contribute to c-Fos expression pattern.

### Conflict of interest statement

The authors declare that the research was conducted in the absence of any commercial or financial relationships that could be construed as a potential conflict of interest.
